# Comparison of Zebrafish *tmem88a* mutant and morpholino knockdown phenotypes

**DOI:** 10.1371/journal.pone.0172227

**Published:** 2017-02-13

**Authors:** Alexander M. J. Eve, Elsie S. Place, James C. Smith

**Affiliations:** Francis Crick Institute, Mill Hill Laboratory, London, United Kingdom; Deakin School of Medicine, AUSTRALIA

## Abstract

Tmem88a is a transmembrane protein that is thought to be a negative regulator of the Wnt signalling pathway. Several groups have used antisense morpholino oligonucleotides in an effort to characterise the role of tmem88a in zebrafish cardiovascular development, but they have not obtained consistent results. Here, we generate an 8 bp deletion in the coding region of the *tmem88a* locus using TALENs, and we have gone on to establish a viable homozygous *tmem88a*Δ8 mutant line. Although *tmem88a*Δ8 mutants have reduced expression of some key haematopoietic genes, differentiation of erythrocytes and neutrophils is unaffected, contradicting our previous study using antisense morpholino oligonucleotides. We find that expression of the *tmem88a* paralogue *tmem88b* is not significantly changed in *tmem88a*Δ8 mutants and injection of the *tmem88a* splice-blocking morpholino oligonucleotide into *tmem88a*Δ8 mutants recapitulates the reduction of erythrocytes observed in morphants using o-Dianisidine. This suggests that there is a partial, but inessential, requirement for *tmem88a* during haematopoiesis and that morpholino injection exacerbates this phenotype in *tmem88a* morpholino knockdown embryos.

## Introduction

The requirement for Wnt signalling in haemovascular development in vertebrates is complex, with activation of the Wnt pathway capable of both promoting and of inhibiting haematopoiesis. Early *in vitro* studies showed that overexpression of β-catenin increased proliferation of haematopoietic stem cells (HSCs), whereas use of Wnt inhibitors prevented HSC growth and reduced their ability to reconstitute the haematopoietic system when transplanted into irradiated mice [1]. Subsequently, conditional expression of constitutively active *β-catenin*, specifically in HSCs, caused a transient expansion of the HSC pool, but it did so at the expense of self-renewal and differentiation, resulting in blood cell depletion and death [2]. Furthermore, HSCs expressing a stable form of β-catenin failed to develop into downstream erythromyeloid lineages and they lost repopulation activity [3].

*In vitro* analyses of mouse HSCs with different hypomorphic mutations in *Apc* show that these cells have increased Wnt levels, increased rates of differentiation, and reduced proliferation [4]. Similar results were obtained in *Wnt3a*^*-/-*^ mice, where HSCs were significantly fewer in number, with poor self-renewal and repopulation potential [5]. In contrast, the non-canonical Wnt ligand Wnt5a enhances self-renewal and promotes quiescence of HSCs, and this is thought to occur by interfering with the ability of Wnt3a to activate the canonical pathway [6]. Indeed, this might be conserved in mammals because human HSCs transplanted into irradiated mice had a greater reconstitution capacity when treated with Wnt-5a conditioned medium [7].

Together, these data suggest that dynamic regulation of canonical and non-canonical Wnt signalling is necessary to balance both blood cell expansion and differentiation. However, we note that many of these studies make use of exogenous activation of the Wnt pathway and the way in which Wnt signalling is modulated during haematopoiesis *in vivo* is poorly understood.

Transmembrane protein 88 (TMEM88; ENSG00000167874) is a two-transmembrane protein with a valine-tryptophan-valine (VWV) motif at the C-terminus that binds the PDZ domain of dishevelled (Dvl). In HEK293 cells, RNAi knockdown of *TMEM88* increased Wnt activity, while overexpression of *TMEM88* attenuated Wnt1-induced activation. A *Siamois* reporter in *Xenopus* showed that TMEM88 inhibits Wnt activation by *xDvl*, also suggesting that TMEM88 negatively regulates Wnt signalling [8].

The zebrafish orthologue, *tmem88a* (ENSDARG00000056920), is expressed in the heart fields, vasculature, and blood islands. This suggests that it might regulate Wnt signalling in these tissues and thereby influence their development *in vivo* [9,10]. Three studies have explored this question. Our own work has shown that *tmem88a* is enriched in *fli1a*:*gfp*^*+ve*^ endothelial cells between 26–28 hpf, and morpholino oligonucleotide (MO) knockdown of *tmem88a* inhibited primitive blood development at 48 hpf [9].

Novikov and Evans showed that *tmem88a* is expressed downstream of *gata5/6* in the heart field but is expressed independently of *gata5/6* in the posterior blood island. MO knockdown of *tmem88a* reduced expression of *nkx2*.*5* in the heart field at the 8-somite stage (8ss), reduced the expression of cardiomyocyte markers at the 23-somite stage, and reduced the number of cardiomyocytes in *Tg(myl7*:*DsRed2-nuc)* embryos at 48 hpf. Heat-shock inducible expression of the Wnt antagonist *dikkopf 1* (*dkk1*) was sufficient to rescue expression of *nkx2*.*5* and to restore the number of *myl7*:*DsRed2*^*+ve*^ cardiomyocytes. In addition, overexpression of full-length *tmem88a* reduced *nkx2*.*5* expression in cardiac progenitors and this expression was rescued by heat-shock inducible *wnt8* expression. This study also observed a significant reduction in *gata1* and *spi1* expression in embryos at the 8-somite stage, further suggesting a requirement for *tmem88a* in primitive haematopoiesis [10].

Most recently, Musso and colleagues showed that the hearts of Tmem88a-deficient zebrafish embryos had increased ventricular conduction velocity at 48 hpf, although the decrease in the number of ventricular nuclei reported by Novikov and Evans was not observed. In addition, Musso and colleagues report the up-regulation of erythrocyte marker *hbbe3* expression in *tmem88a* knockdown embryos, in contrast to the down-regulation of *hbbe1* observed in our own experiments. Finally, the development of cyclopia in embryos lacking Wnt11 was exacerbated when *tmem88a* was also knocked down [11]. These data suggest a role for Tmem88a in regulating the non-canonical Wnt cascade, either directly or indirectly by inhibiting canonical Wnt activation [12].

The different results obtained by different groups might be explained in part by off-target effects of anti-sense morpholino oligonucleotides. Indeed, several studies have described variation between the phenotypes of MO knockdown and genetic mutants [13–20]. To define definitively the role of *tmem88a* in zebrafish haematopoiesis, we therefore made a genetic mutant using transcription activator-like effector nucleases (TALENs). We show that the mutants are morphologically normal and viable, but have reduced expression of some genes associated with early cardiovascular development. We also describe possible off-target effects of morpholino injection that appear to exacerbate the phenotype in MO knockdown embryos, but not in mutants.

## Materials and methods

### Protein alignments

Human, *Xenopus*, and zebrafish TMEM88 protein sequences were aligned using EMBL-EBI Clustal Omega software. Phylogenetic tree data was organised into an unrooted phylogenetic tree cladogram using the TreeDraw software from the PHYLIP package [21,22].

### Ethics statement

All zebrafish work was carried out with approval from the Francis Crick Institute Biological Research Facility Strategic Oversight Committee and the Animal Welfare and Ethical Review Body, and in accordance with the Animals (Scientific Procedures) Act 1986, the Animal Welfare Act (2006) and the Welfare of Animals in Transport Order. Care was taken to minimize the numbers of animals used in these experiments in accordance with the ARRIVE guidelines (http://www.nc3rs.org.uk/page.asp?id=1357).

### Zebrafish stocks and maintenance

Zebrafish (*Danio rerio*) adults were maintained and bred under standard conditions [23] and embryos were staged as described previously [24]. The wild-type (WT) Lon AB line was provided by the Francis Crick Institute, Mill Hill Laboratory aquatics facility (London, UK). The published transgenic line *Tg(fli1a*:*egfp)*^*y1*^ [25] was a gift from Dr Tim Chico (MRC Centre for Developmental and Biomedical Genetics, University of Sheffield). The *Tg(fli1a*:*egfp)*^*tmem88aΔ8*^ (“*tmem88a*Δ8”) strain was created as part of this work.

### TALEN synthesis and mutagenesis

Two TALENs were designed to target exon 2 of *tmem88a*, either side of a spacer region containing a ScaI endonuclease site. TALEN constructs were assembled from the Golden Gate TALEN 2.0 plasmid kit, a gift from Daniel Voytas and Adam Bogdanove (Addgene, Kit #1000000024), as previously described and according to the manufacturer’s protocol [26,27]. Complete TALEN plasmid DNA was linearised with SacI (New England BioLabs) in a 50 μl reaction and purified using QIAquick purification columns (Qiagen) according to the manufacturer’s instructions. mRNA was synthesised from linear plasmid using the T3 mMESSAGE mMACHINE kit (Ambion, Life Technologies) according to the manufacturer’s instructions. It was purified by LiCl precipitation. 1 nl containing 60 ng/nl of each TALEN mRNA was injected into single-cell *Tg(fli1a*:*egfp)*^*y1*^ embryos.

### Genotyping

To extract genomic DNA, single 24 hpf embryos or adult tail fin clips were incubated in 10–50 μL extraction buffer containing 50 mM Tris-HCl (pH 8.5), 1 mM EDTA, 0.5% Tween-20 and 80 mg/ml proteinase K (Ambion, Life Technologies) for 3 hours at 55°C. Proteinase K was inactivated by heating to 95°C for 10 minutes. 1 μl of genomic DNA mix was used in a standard 50 μl PCR reaction with Phusion High-Fidelity DNA polymerase (New England BioLabs) according to the manufacturer’s instructions and using primers spanning the TALEN target site (Fw: 5′-CACACAGCCCAATGCATGAC-3′; Rv: 5′-TCTTTTTCCTTGGCATTGGGTA-3′). PCR products were purified using QIAquick purification columns (Qiagen) and digested overnight at 37°C in a 10 μl reaction containing ScaI-HF endonuclease (New England BioLabs) according to the manufacturer’s instructions. Samples were then run on a 2% agarose gel. Mutant products were undigested at 705 bp, whereas digested wild-type products were detected as fragments of 240 and 465 bp.

Genotypes were also sequence verified using 1μl of genomic DNA in a standard 50 μl PCR reaction with Phusion High-Fidelity DNA polymerase (New England BioLabs) according to the manufacturer’s instructions, and using primers spanning the TALEN target site: primers include the SP6 promoter sequence, which is shown underlined; Fw: 5′–ATTTAGGTGACACTATAGAAGNGAAAACTGGCCCTTCACATAC-3′; Rv: 5′– AATGGCAGAGGAAGCCAAAAAC-3′. PCR products were purified using QIAquick purification columns (Qiagen) according the manufacturer’s instructions, sequenced using SP6 promoter, and aligned to the wild type genomic sequence.

### Reverse transcription, quantitative RT-PCR and melting curve analyses

Between 30 and 50 embryos were collected for extraction and purification of RNA using TRIzol and RNeasy Minikit Columns (Qiagen) according to the manufacturer’s protocol. cDNA was reverse transcribed from 0.5–3 μg total RNA using M-MLV RT RNase (-H) Point Mutant (Promega) according to the manufacturer’s instructions and diluted 1:10 for qRT-PCR. Quantitative RT-PCR was performed in duplicate 10 μl reactions using Lightcycler Mastermix (Roche) on a Lightcycler LC480 (Roche) according to the manufacturer’s instructions. Primer sequences were as previously published or designed using NCBI primer-BLAST and are shown in Table 1 [9]. Two primer sets detecting all *tmem88a* transcript (F1R1) and only wild type *tmem88a* transcript (F2R2) were used. Expression levels were compared to a standard curve, normalised to total RNA, and expressed as a percentage of the control groups (defined as 100%). Melt curves using cDNA synthesised from RNA extracted from *Tg(fli1a*:*egfp)* and *tmem88*Δ8 mutant embryos in biological triplicate using Light Cycler 480 software version 1.5.0.

**Table 1:** Sequences of primers used for qRT-PCR.

	Forward primer (5′-3′)	Reverse primer (5′-3′)
*fli1a*	AGCGCTACGCCTACAAGTTC	AGCTCCAGTATGGGGTTGTG
*gata1a*	CTCGTTGGGTGTCCCCCGGT	CGACGAGGCTCGGCTCTGGA
*hbbe1*	AACTGTGCTCAAGGGTCTGG	TACGTGGAGCTTCTCGGAGT
*lyz*	GCACGGCCTACTGGGAAAGCA	CCCAGGGGTCCCGTCATCACA
*myoD*	GGGCCCAACGTGTCAGACGA	GTTGAGGGCAGCTGGTCGGG
*nkx2*.*5*	CTGTGCCAGTTTTGGTTCGG	CGCAGGGTAGGTGTTGTAGG
*runx1*	AGTGGACGGACCCCGAGAGC	ACCGCATGGCACTTCGCCTC
*scl*	CAACGATGGTTCGCAGCCCA	ACCGCCGACCATGTCGTCCT
*spi1b*	ATGCGGCCAGTGTGCATCGC	CACCGATGTCCGGGGCAAGT
*tmem88a* F1R1	CCTGCCATCGCTCGTCATGGT	AGACGGCACGGCTGTATGGGA
*tmem88a* F2R2	TGCCAGTCTGAGGAATCTGCC	GTTTGCTGGAGTACTGGAGGAGA
*tmem88b*	CCTCCACCTTACTCCCCAGA	ATGGGATAAAGCACAGCCCC

*fli1a*, *friend leukaemia integration 1a*; *gata1a*, *GATA-binding factor 1a*; *hbbe1*, *haemoglobin beta-embryonic 1*; *lyz*, *lysozyme*; *runx1*, *runt-related transcription factor 1*; *scl*, *stem cell leukaemia*; *spi1*, *spleen focus forming virus* (*sffv*) and *proviral integration oncogene 1b* (also known as *pu*.*1*).

For digestion of *tmem88a* cDNA, 2 μl of cDNA synthesised from wild type and *tmem88a* mutant RNA was used in a standard 20 μl PCR reaction with Phusion High-Fidelity DNA polymerase (New England BioLabs) according to the manufacturer’s instructions. The primers used spanned the deletion and contained a 5′ SP6 promoter sequence, which is shown underlined (Fw: 5′–ATTTAGGTGACACTATAGAAGNGAAAACTGGCCCTTCACATAC-3′; Rv: 5′– AATGGCAGAGGAAGCCAAAAAC-3). 10 μl of each PCR product was digested overnight at 37°C with ScaI endonuclease (Thermo Fisher Scientific) according to the manufacturer’s instructions. Samples were then run on a 2% agarose gel. Mutant products were undigested at 423 bp, whereas digested wild type products were detected as fragments of 244 and 179 bp.

### DIG-labelled probe synthesis and whole mount *in situ* hybridisation

Anti-sense *fli1a* probes were synthesised as previously described [9]. Antisense DIG-labelled probes for *gata1a* were synthesised using the SP6 promoter and cDNA template amplified using the following primers: the SP6 promoter sequence is underlined and the T7 promoter sequence is shown in italics; Fw: 5′-*TAATACGACTCACTATAG*GGAGACACTCTCACACCTCCACGTC-3′; Rv: 5′-ATTTAGGTGACACTATAGAAGNGATTGAGGGAAACAAAAAGTGTGT-3′. DIG-labelled anti-sense *myoD* probes were synthesised from the T7 promoter and template plasmid DNA (IRBOp991B1179D, Source Bioscience) linearised with EcoRV according to the manufacturer’s instructions. *In situ* hybridisation was performed as previously described [28,29].

### Whole embryo staining for blood cells

To study erythrocyte development, o-Dianisidine staining was performed on 48 hpf embryos as previously described [30]. Stained embryos were then fixed in 4% paraformaldehyde in PBS (Thermo Scientific) for 20 minutes at room temperature, extensively rinsed in PBST, and mounted for imaging.

To investigate neutrophil development, embryos were fixed for 1 hour at room temperature in 4% glutaraldehyde (Sigma-Aldrich) in borate buffer. Embryos were then stained with Sudan Black B (Sigma-Aldrich): 0.18% (w/v) Sudan Black, in 69% ethanol; for 20 minutes at room temperature with agitation. Embryos were then extensively rinsed in three times in 70% EtOH with agitation, and rehydrated in PBST.

### Mounting, imaging and image analysis

For imaging, stained embryos were mounted in 50% glycerol on glass slides with coverslips. 8-somite stage (8ss) embryos were flat mounted as previously described [29]. Whole embryos were imaged using a Leica M165 FC microscope and Leica Application suite 3.4.1 software. o-Dianisidine staining was quantified by dividing the stained area of the yolk by the total yolk area using Adobe Photoshop and Image J software, and presented as a percentage of the yolk. All quantification of blood cells was performed blind.

### Morpholino oligonucleotide (MO) knockdown

One-cell *Tg(fli1a*:*egfp)*^*y1*^ or *tmem88a*Δ8 embryos were injected with 5 ng of both *tmem88a* e2i2 splice-blocking MO (SBMO; 5′-GCATTCTCACTCCACACATACCGTT-3′) and *p53* MO (5′-GCGCCATTGCTTTGCAAGAATTG-3′) as previously described [9].

## Results

### TALEN mutagenesis of *tmem88a*

**Fig 1:**
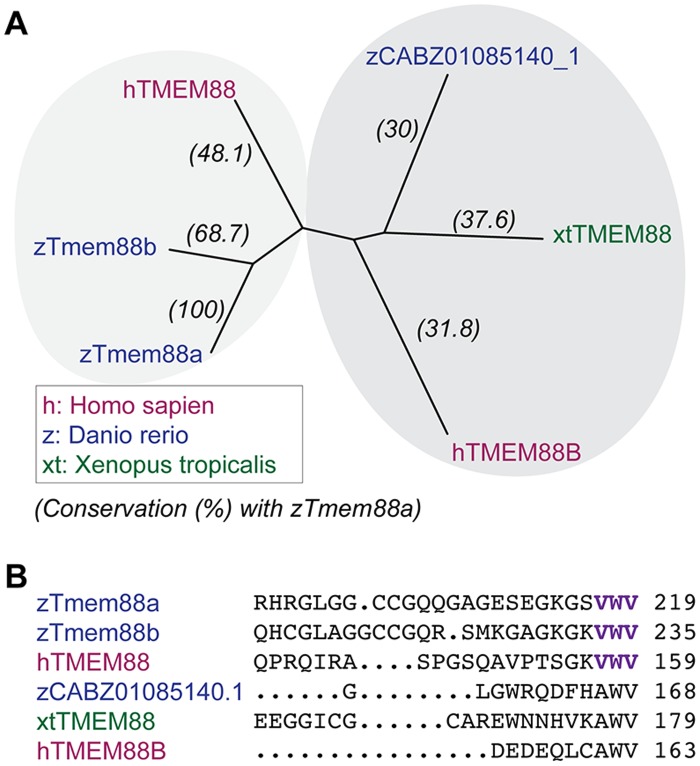
Species conservation of TMEM88 proteins. (A) Unrooted phylogenetic tree of aligned TMEM88 protein orthologues from human (*Homo sapiens*, magenta), *Xenopus tropicalis* (green), and zebrafish (*Danio rerio*, blue) show clustering into two branches of sequence conservation. (B) Zebrafish orthologues—Tmem88a and Tmem88b –share the conserved valine-tryptophan-valine (VWV) motif in the Dishevelled (Dvl) binding domain of human TMEM88.

Human (*Homo sapiens*), *Xenopus tropicalis*, and zebrafish (*Danio rerio*) TMEM88 protein sequences were aligned using Clustal Omega and grouped in an unrooted phyologenetic tree based on sequence conservation. The results reveal two main clusters, the first of which includes human TMEM88 and the zebrafish orthologues Tmem88a and Tmem88b (ENSDARG00000069388) (Fig 1A). The zebrafish Tmem88a and Tmem88b proteins share the valine-tryptophan-valine (VWV) motif, which was previously identified as being involved in Dishevelled binding [8]. Members of the second cluster are less closely related and include human TMEM88B (ENSG00000205116), *Xenopus* TMEM88 (NW_004668240.1), and the zebrafish orthologue CABZ01085140.1 (ENSDARG00000098070), which all lack the conserved VWV motif (Fig 1B).

**Fig 2:**
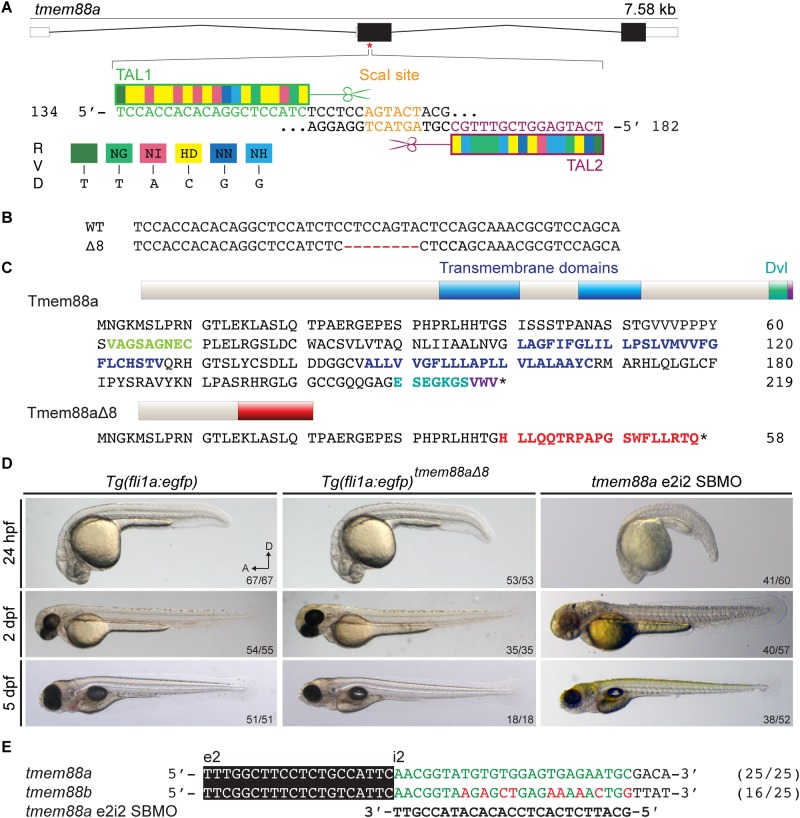
Mutation of the zebrafish *tmem88a* gene. (A) Schematic structure of the zebrafish *tmem88a* gene shows three exons and two introns. TALEN pairs TAL1 (green) and TAL2 (magenta) were designed and assembled to target the coding region of exon2. There is a ScaI endonuclease restriction site within the spacer region (orange). (B) A *tmem88a* mutant line with an 8 bp deletion (Δ8) was generated. (C) Predicted translated sequences of wild-type Tmem88a and Tmem88aΔ8 show frame-shift after residue 48 (red), causing truncation of the protein and loss of the trans-membrane domains (blue) and the Dishevelled-binding domain (teal). (D) Brightfield images of control *Tg(fli1a*:*egfp)* and *tmem88a*Δ8 embryos at 24 hpf (n = 67, n = 53 respectively), 2 dpf (n = 54, n = 35), and 5 dpf (n = 51, n = 18) as labelled. (E) Sequence alignment shows that the *tmem88a* e2i2 SBMO is specific to *tmem88a* and illustrates a 9 bp mismatch with *tmem88b* mRNA.

TALENs were designed to target the coding region in exon2 of the *tmem88a* locus. This target included a ScaI restriction site that would be destroyed by mutations in the spacer region. ScaI digestion was used for genotyping TALEN-injected embryos and subsequent generations of fish (Fig 2A). TALEN constructs were injected into *Tg(fli1a*:*egfp)* embryos. F_0_ adults were screened for *tmem88a* mutations by PCR amplification of genomic DNA followed by ScaI digestion. An F_0_ founder female was crossed with a wild type Lon AB male to produce an F_1_ generation. The F_1_ generation was in-crossed and F_2_ adults were screened for mutations by sequencing the target region. F_2_ mutants were in-crossed and F_3_ adults with homozygous deletions were selected by sequencing to produce a homozygous mutant line, with an 8 bp deletion at position 120 of the coding sequence, expected to cause a frame-shift and nonsense-mediated decay of the transcript (Fig 2B). The translated sequence of *tmem88a*Δ8 was predicted to include an early stop codon and truncation of the protein, preventing translation of the transmembrane and Dishevelled-binding domains (Fig 2C).

To our surprise, *tmem88a*Δ8 mutant embryos showed no morphological abnormalities during early development (0–5 dpf), and did not exhibit any developmental defects when compared with *Tg(fli1a*:*egfp)* controls of the same genetic background and no developmental delay was observed compared to *tmem88a* SBMO knockdown embryos (Fig 2D). Indeed, the line proved to be viable and *tmem88a*Δ8 mutants produced fertile adults. Sequence alignments show morpholino specificity for *tmem88a* transcripts and not double knockdown of *tmem88a* and *tmem88b* because of a 9 bp mismatch (Fig 2E) [31].

### Wild type *tmem88a* transcripts are absent in *tmem88a*Δ8 mutant embryos

**Fig 3:**
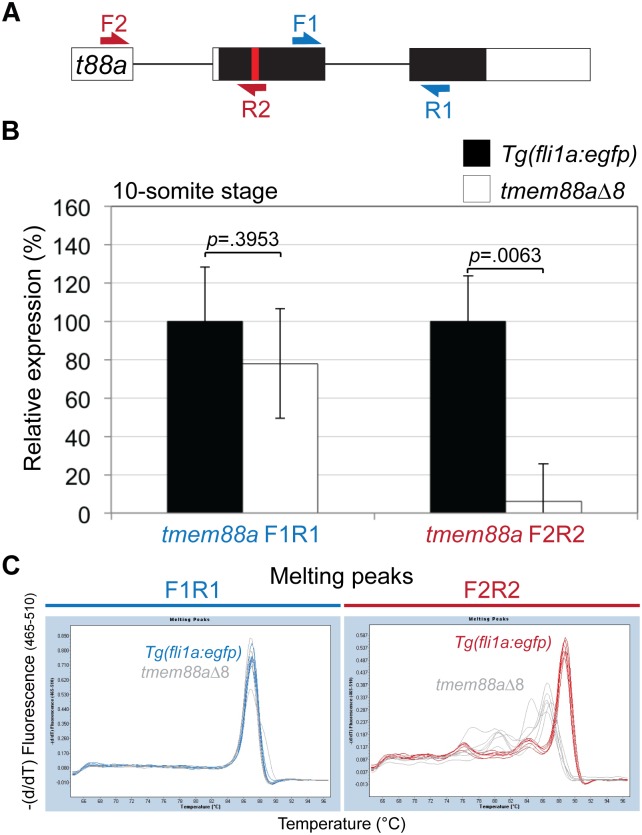
The expression of *tmem88a* in *tmem88*Δ8 mutant embryos. (A) Schematic representation of the *tmem88a* (*t88a*) mRNA transcript. Non-coding and coding exons are shown as white and black blocks respectively. Introns as depicted as solid lines. The deleted site in *tmem88a*Δ8 mutants is shown by a red bar. F1R1 primers detected tmem88a transcript and F2R2 primers detected wild type transcript only. (B) qRT-PCR showing the expression of *tmem88a* in *tmem88*Δ8 mutants compared to *Tg(fli1a*:*egfp)* controls at the 10-somite stage using both primer sets. *P*-values determined by Unpaired Student’s t test. Error bars show the standard deviation for three biological replicates. (C) Melting curves using two primer sets designed for *tmem88a* detection as shown in (A). A shift in melting in temperature is observed in primers designed to bind the mutated region in *tmem88a*Δ8 mutants compared to controls (F2R2, green).

**Fig 4:**
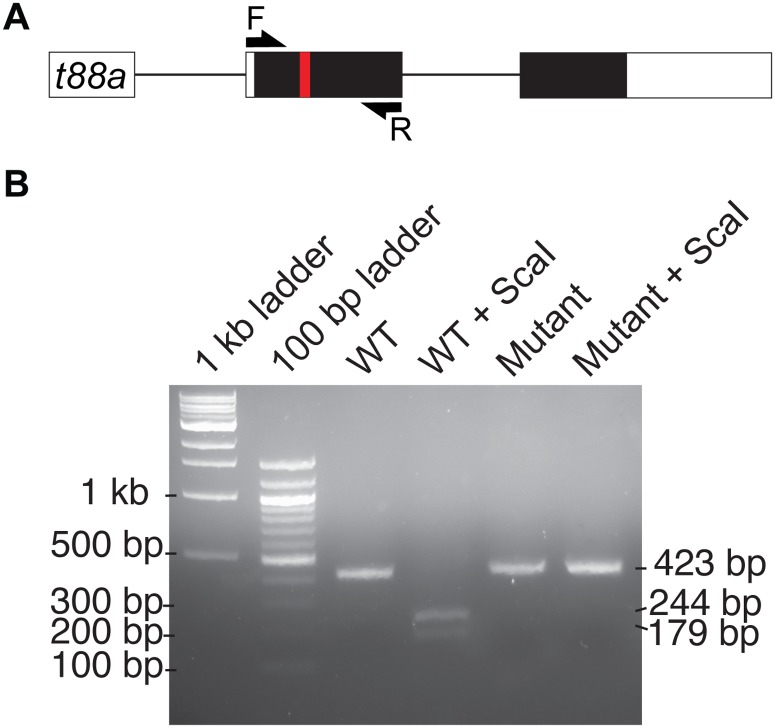
*tmem88a* cDNA from *tmem88a* mutants is not digested by ScaI. (A) Schematic representation of the *tmem88a* (*t88a)* mRNA transcript. Non-coding and coding exons are shown as white and black blocks respectively. Introns as depicted as solid lines. The deleted site in *tmem88a*Δ8 mutants is shown by a red bar. F and R primers used for amplification of cDNA are shown as arrows. (B) 2% agarose gel showing ScaI digestion of wild type PCR product into fragments of 244 and 179 bp in size. *Tmem88a* mutant cDNA is not digested by ScaI.

To validate knock out of the *tmem88a* gene qRT-PCR was performed using RNA recovered from 10-somite stage embryos. Two primer sets were used as illustrated, F1R1 detected all spliced *tmem88a* transcript, while F2R2 only detects wild type *tmem88a* transcript because the reverse primer binds across the mutated region (Fig 3A). Results do not show a significant reduction (*p* = 0.3953, Unpaired Student’s t test) in *tmem88a* transcript in *tmem88a*Δ8 mutants compared to controls, suggesting that not all transcripts were degraded by nonsense-mediated decay (Fig 3B). However, a significant decrease (*p* = 0.0063, Unpaired Student’s t test) in wild type *tmem88a* transcript was found in *tmem88a*Δ8 embryos compared to controls (Fig 3B). Analyses of the melting curves generated by the two primers sets showed a shift in F2R2 primers when binding mutant cDNA, suggesting no wild type transcripts were detected (Fig 3C). In addition, cDNA generated from *tmem88a* mutants is not digested by ScaI endonuclease compared with wild type controls, providing further evidence that wild type transcripts are not present in *tmem88a* mutant embryos (Fig 4).

### Reduced expression of some key cardiac and haematopoietic factors is observed in *tmem88a* mutant embryos

**Fig 5:**
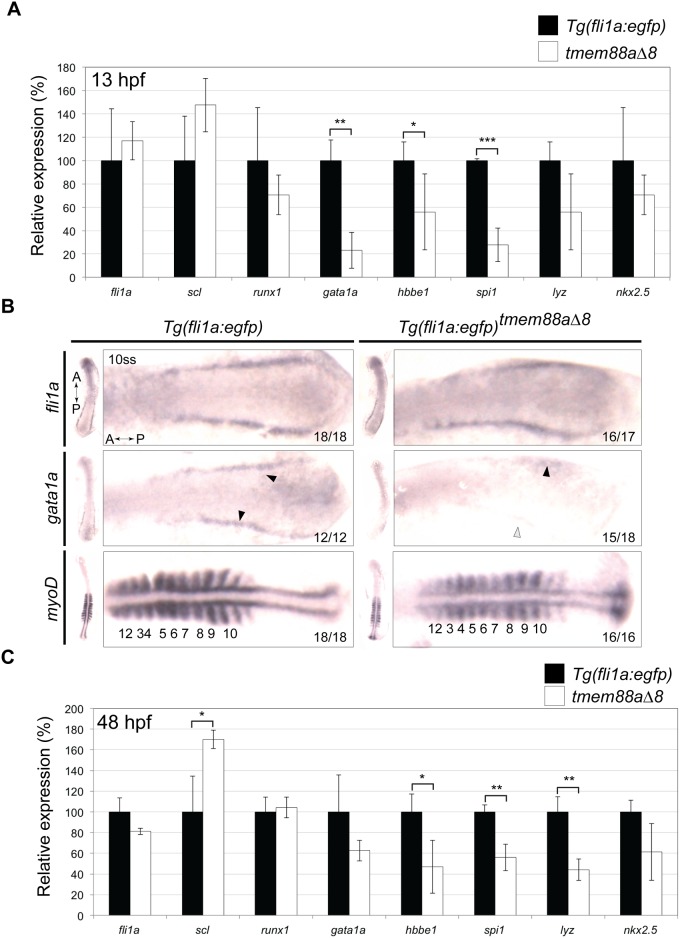
Cardiovascular associated genes are downregulated in *tmem88a*Δ8 embryos. (A) qRT-PCR showing the expression of key cardiovascular genes in *tmem88*Δ8 mutants compared to *Tg(fli1a*:*egfp)* controls at 13 hpf. (B) *In situ hybridisation* for *fli1a*, *gata1a*, and *myoD* in control and *tmem88a*Δ8 mutants as labelled. *Gata1a* expression is denoted with black error heads, reduced or absent expression is shown with grey or white arrowheads respectively. Somites are marked by asterisks. (C) qRT-PCR showing the expression of key cardiovascular genes in *tmem88*Δ8 mutants compared to *Tg(fli1a*:*egfp)* controls at 48 hpf. All *P*-values determined by Unpaired Student’s t test. Error bars denote the standard deviation of three biological replicates. *P*-values determined by Unpaired Students t test. * = *p*<0.05; ** = *p*<0.01; *** = *p*<0.001; unlabelled bars show no significant difference (*p*>0.05). *fli1a*, *friend leukaemia integration 1a*; *gata1a*, *GATA-binding factor 1a*; *hbbe1*, *haemoglobin beta-embryonic 1*; hpf, hours post fertilisation; *lyz*, *lysozyme*; *runx1*, *runt-related transcription factor 1*; *scl*, *stem cell leukaemia*; *spi1*, *spleen focus forming virus* (*sffv*) and *proviral integration oncogene 1b* (also known as *pu*.*1*).

*Tmem88a* knockdown has previously been shown to reduce expression of haematopoietic genes [9,10]. We therefore investigated the expression of known cardiovascular markers in *tmem88a*Δ8 embryos. *Tg(fli1a*:*egfp)* and *tmem88*Δ8 embryos were collected at 13 hpf, and RNA extracted for qRT-PCR. Although there was no significant difference between the expression of *fli1a*, *scl*, and *runx1* at this stage, there was a significant (p<0.05, Unpaired Student’s t test) reduction in *gata1a*, *hbbe1*, and *spi1* expression (Fig 5A). To confirm this observation we performed *in situ* hybridisation on 10-somite stage embryos. There was no gross difference in *fli1a* expression between the two lines (controls: 18/18; *tmem88a*Δ8 mutants: 16/17), but *gata1a* expression was greatly reduced in *tmem88a*Δ8 embryos (15/18) compared to controls (12/12). Expression of *MyoD*, studied as a control for developmental delay, was slightly weaker in mutant embryos, with expression varying a little between somites (Fig 5B).

### *tmem88a* is dispensable for primitive haematopoiesis

**Fig 6:**
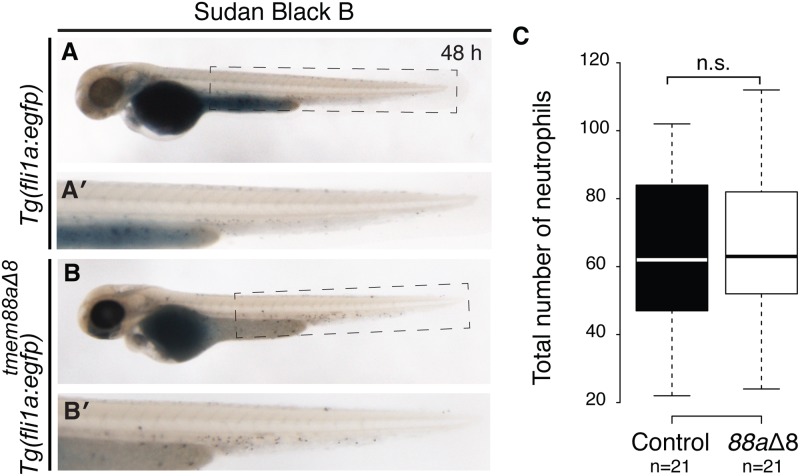
Myelopoiesis was not affected in *tmem88a*Δ8 zebrafish mutants. (**A-B**) Sudan Black B staining of neutrophils at 48 hpf in *Tg(fli1a*:*egfp)* controls (A, n = 21), with the highlighted tail region magnified (A′) and *tmem88a*Δ8 mutants (B, n = 21), magnified in (B′). (**C**) The number of neutrophils was counted for each condition represented in a Tukey box and whisker plot. Neutrophil numbers were not significantly changed between the two groups (*p* = 0.6769, Unpaired Student’s t test). A, anterior; h, hours post fertilisation; D, dorsal; n.s., not significant.

*Tmem88a*Δ8 mutants showed a reduction of myeloid markers *spi1b* at 13 and 48 hpf, and *lyz* at 48 hpf. However, *tmem88a*Δ8 mutants were otherwise unaffected and survived to adulthood, suggesting that this reduction was not sufficient to prevent blood development. To investigate whether the myeloid cell lineage was affected, 48 hpf *Tg(fli1a*:*egfp)* control embryos (n = 21) and *tmem88a*Δ8 mutants (n = 21) were stained for neutrophils with Sudan Black B solution (Fig 6A and 6B). Neutrophils were counted and results showed no significant difference (*p* = 0.6769, Unpaired Student’s t test) between the number of cells in control embryos and Tmem88a-deficient embryos (Fig 6C). This suggests that *tmem88a* is not required for primitive myelopoiesis.

**Fig 7:**
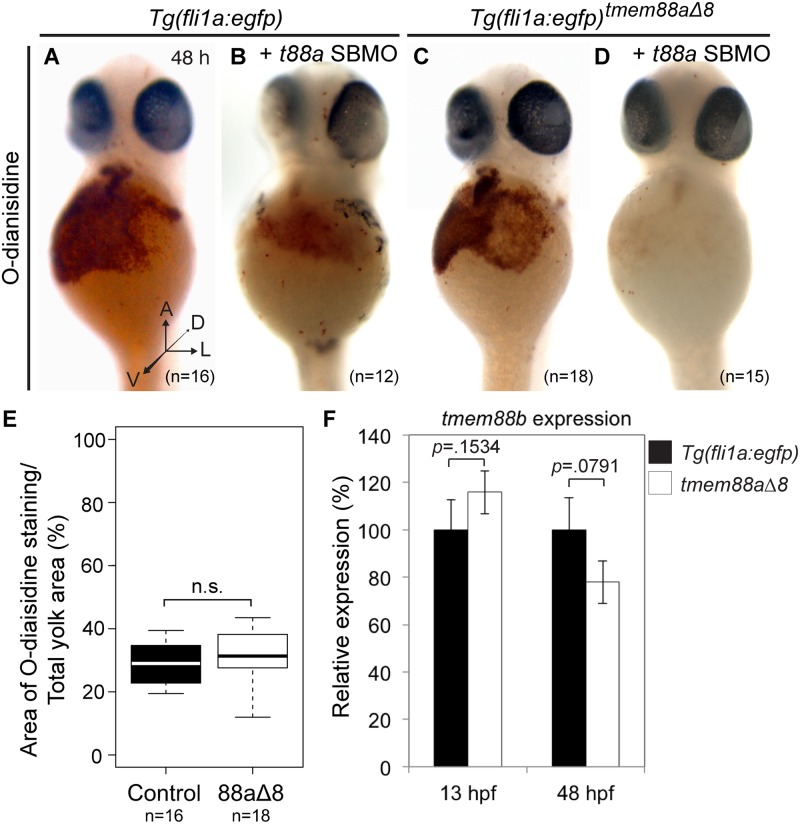
Erythropoiesis was not affected in *tmem88a*Δ8 zebrafish mutants. (**A-D**) o-Dianisidine staining of erythrocytes at 48 hpf in uninjected *Tg(fli1a*:*egfp)* controls (A, n = 16) or controls injected with *tmem88a* SBMO (B, n = 12), and uninjected *tmem88a*Δ8 mutants (C, n = 18) or mutants injected with *tmem88a* SBMO (D, n = 15). (**E**) The area of o-Dianisine staining was quantified and presented as a percentage of the total yolk area, shown in a Tukey box and whisker plot. No significant difference was found between control embryos and *tmem88a*Δ8 mutants (*p* = 0.2710, Unpaired Student’s t test). (**F**) *tmem88b* expression is not affected in *tmem88a*Δ8 mutants. qRT-PCR showing the expression of *tmem88b* in *tmem88*Δ8 mutants compared to *Tg(fli1a*:*egfp)* controls at 13 and 48 hpf. *P*-value determined by Unpaired Student’s t test. Error bars denote the standard deviation of three biological replicates. A, anterior; h, hours post fertilisation; D, dorsal; L, lateral; V, ventral; n.s., not significant.

We also observed a reduction in expression of erythrocyte markers *gata1a* and *hbbe1* in *tmem88a*Δ8 mutants at 13 and 48 hpf. To ask whether primitive erythropoiesis was affected, we performed o-Dianisidine staining on *Tg(fli1a*:*egfp)* control (n = 16) and *tmem88a*Δ8 embryos (n = 18) at 48 hpf (Fig 7A and 7C). Staining was quantified by measuring the area of stained cells as a percentage of the yolk (Fig 7E). There was no significant difference between *Tg(fli1a*:*egfp)* controls and Tmem88a-deficient embryos (*p* = 0.2710, Unpaired Student’s t test).

These results contrast with our previous observations that both myelocyte peroxidase and o-Dianisidine staining was reduced when *tmem88a* was knocked-down using MOs. It is possible that the differences between the mutant and morphant phenotypes reflect genuine biological differences relating to the effects of genetic knockout versus knockdown by interfering oligonucleotides. One recent study has suggested that compensatory transcriptional changes may occur when a gene is inactivated by genetic mutation, but not as a result of knockdown in morphants [14]. One way in which compensation could occur is through the upregulation of a paralogue. We therefore examined the expression of the *tmem88a* paralogue, *tmem88b*, in the *tmem88a* mutant line and did not see a significant change in *tmem88b* expression (Fig 7F).

Next, we next asked whether the loss of blood cells seen in morphant embryos was due to an off-target effect of MO injection. We injected the *tmem88a* splice-blocking MO (SBMO) and a *p53* MO into *Tg(fli1a*:*egfp)* controls (n = 12) and *tmem88a*Δ8 embryos (n = 15), on the basis that *tmem88a*Δ8 embryos lack wild-type tmem88a protein, and thus any defects seen cannot be due to loss of protein. *Tmem88a* SBMO injection caused a clear reduction in o-Dianisidine staining of erythrocytes in both control and in *tmem88a*Δ8 mutants (Fig 7B and 7D). This suggested that off-target effects from morpholino injection affected o-Dianisidine staining of erythrocytes.

## Discussion

The role of *tmem88a* in zebrafish development has been investigated by three groups using antisense morpholino oligonucleotides. All identify cardiovascular phenotypes, but otherwise the results have been inconsistent [9–11,32]. To resolve this issue we generated an 8 bp deletion and frame-shift mutation in the endogenous *tmem88a* locus using TALEN technology [26]. In this way we generated a viable *tmem88a* loss-of-function zebrafish mutant line to study the role of *tmem88a* in primitive haematopoiesis.

### *tmem88a* is dispensable for zebrafish haematopoiesis

*In situ* hybridisation and qRT-PCR revealed that the expression of key haematopoietic factors *gata1a*, *hbbe1*, and *spi1* are reduced in 8-somite stage and 48 hpf *tmem88a*Δ8 mutants. This is consistent with *tmem88a* morpholino knockdown data, which also showed a reduction of haematopoietic markers at these time points [9,10]. Bearing in mind the probable role of *tmem88a* as a regulator of Wnt signalling, these observations suggest that temporal and spatial Wnt inhibition is necessary to promote haematopoiesis [8]. Although we observed that *tmem88a*Δ8 mutants had reduced expression of haematopoietic markers, equivalent to the reduction seen by *tmem88a* morpholino knockdown [9], this was not sufficient to prevent the development of mature primitive erythrocytes and neutrophils as determined by histological staining. This suggests that although haematopoietic genes are down-regulated in the absence of Tmem88a, primitive erythrocytes and neutrophils still formed. Thus, there is a partial, but not essential, requirement for *tmem88a* in primitive blood cell development.

### Differences between mutation and knockdown

Our mutant data conflict with our previously-described morpholino knockdown phenotype, in which, according to o-Dianisidine staining and myelocyte peroxidase assays, the numbers of primitive erythrocytes and neutrophils were reduced [9]. No significant morphological heart defects, like those described by Novikov and Evans, were observed in *tmem88a*Δ8 mutants [10]. We did not observe a significant decrease in *nkx2*.*5* expression in *tmem88a*Δ8 mutants using qRT-PCR, although we note that our study of cardiac factors was limited.

There have been several examples of differences between MO knockdown and genetic mutant phenotypes, and these differences have been suggested to represent nonspecific off-target effects of morpholinos [13,16–20,33–35]. In some cases mutant alleles may not represent true loss-of-function phenotypes because use of alternate (non-AUG) translation start codons, cryptic splice sites, or translational readthrough prevent complete loss of the target protein [36–40]. rpholino to it;s they are more toxic effects of morpholinos are induced by the binding of the morpholino to it;s they are more Alternatively, one or several proteins with similar functions might be up-regulated to compensate in genetic mutants, but not in morphants [14]. Although *tmem88b* –an obvious candidate for a compensatory gene in *tmem88a*Δ8 mutants—was not significantly up-regulated compared with controls, there may be up-regulation of other genes that we have not investigated. Injecting a morpholino specific to *tmem88a* into *tmem88a*Δ8 mutants reduced o-Dianisidine staining, suggesting that erythrocyte development was affected by morpholino injection independently of Tmem88a-deficiency. It is known that morpholinos can induce nonspecific toxic effects such as pericardial oedema, reduced cardiomyocyte proliferation, and p53-dependent cell death [31,41–44]. Furthermore, many stressors, including morpholinos, are known to cause heart failure and reduced circulatory flow, which is required for proper vascular development, although the mechanisms by which stressors cause these effects remain unknown [45,46]. Finally, it is possible that in this case the reduced expression of haematopoietic factors in Tmem88a-deficient embryos might increase susceptibly to such stressors, increasing the likelihood of nonspecific vascular abnormalities occurring as a result of morpholino injection.

## Conclusion

We observed that although *tmem88a* mutants had a reduction of some cardiovascular markers equivalent to those seen in *tmem88a* morphants, *tmem88a* was dispensable for primitive haematopoiesis because erythrocytes and neutrophils were identified, respectively, by o-Dianisidine and Sudan Black B staining. Injecting a *tmem88a* MO into mutants reduced o-Dianisidine staining to the level observed in *tmem88a* morphants. This suggests that MO use might have off-target effects on later erythrocyte development and the that published *tmem88a* MO knockdown phenotypes might include such nonspecific toxic effects.

## References

[1] Reya T, Duncan AW, Ailles L, Domen J, Scherer DC, Willert K, et al A role for Wnt signalling in self-renewal of haematopoietic stem cells. Nature. 2003;423: 409–414. 10.1038/nature01593 12717450

[2] Scheller M, Huelsken J, Rosenbauer F, Taketo MM, Birchmeier W, Tenen DG, et al Hematopoietic stem cell and multilineage defects generated by constitutive beta-catenin activation. Nat Immunol. 2006;7: 1037–1047. 10.1038/ni1387 16951686

[3] Kirstetter P, Anderson K, Porse BT, Jacobsen SEW, Nerlov C. Activation of the canonical Wnt pathway leads to loss of hematopoietic stem cell repopulation and multilineage differentiation block. Nat Immunol. 2006;7: 1048–1056. 10.1038/ni1381 16951689

[4] Famili F, Brugman MH, Taskesen E, Naber BEA, Fodde R, Staal FJT. High Levels of Canonical Wnt Signaling Lead to Loss of Stemness and Increased Differentiation in Hematopoietic Stem Cells. Stem Cell Reports. The Authors; 2016;6: 652–659. 10.1016/j.stemcr.2016.04.009 27167156 PMC4939829

[5] Luis TC, Weerkamp F, Naber BAE, Baert MRM, De Haas EFE, Nikolic T, et al Wnt3a deficiency irreversibly impairs hematopoietic stem cell self-renewal and leads to defects in progenitor cell differentiation. Blood. 2009;113: 546–554. 10.1182/blood-2008-06-163774 18832654

[6] Nemeth MJ, Topol L, Anderson SM, Yang Y, Bodine DM. Wnt5a inhibits canonical Wnt signaling in hematopoietic stem cells and enhances repopulation. Proc Natl Acad Sci U S A. 2007;104: 15436–41. 10.1073/pnas.0704747104 17881570 PMC1986571

[7] Murdoch B, Chadwick K, Martin M, Shojaei F, Shah K V, Gallacher L, et al Wnt-5A augments repopulating capacity and primitive hematopoietic development of human blood stem cells in vivo. Proc Natl Acad Sci U S A. 2003;100: 3422–7. 10.1073/pnas.0130233100 12626754 PMC152308

[8] Lee HJ, Finkelstein D, Li X, Wu D, Shi DL, Zheng JJ. Identification of transmembrane protein 88 (TMEM88) as a dishevelled-binding protein. J Biol Chem. 2010/11/04. 2010;285: 41549–41556. 10.1074/jbc.M110.193383 21044957 PMC3009882

[9] Cannon JE, Place ES, Eve AMJ, Bradshaw CR, Sesay A, Morrell NW, et al Global analysis of the haematopoietic and endothelial transcriptome during zebrafish development. Mech Dev. 2013;130: 122–131. 10.1016/j.mod.2012.10.002 23072875 PMC3580284

[10] Novikov N, Evans T. Tmem88a mediates GATA-dependent specification of cardiomyocyte progenitors by restricting WNT signaling. Development. 2013; 140. 10.1242/dev.093567 PMC3754477 23903195

[11] Musso G, Tasan M, Mosimann C, Beaver JE, Plovie E, Carr L a, et al Novel cardiovascular gene functions revealed via systematic phenotype prediction in zebrafish. Development. 2014;141: 224–35. 10.1242/dev.099796 24346703 PMC3865760

[12] Bisson JA, Mills B, Helt JCP, Zwaka TP, Cohen ED. Wnt5a and Wnt11 inhibit the canonical Wnt pathway and promote cardiac progenitor development via the Caspase-dependent degradation of AKT. Dev Biol. Elsevier; 2015;398: 80–96. 10.1016/j.ydbio.2014.11.015 25482987

[13] Kok FO, Shin M, Ni CW, Gupta A, Grosse AS, van Impel A, et al Reverse Genetic Screening Reveals Poor Correlation between Morpholino-Induced and Mutant Phenotypes in Zebrafish. Dev Cell. Elsevier Inc.; 2015;32: 97–108. 10.1016/j.devcel.2014.11.018 PMC4487878 25533206

[14] Rossi A, Kontarakis Z, Gerri C, Nolte H, Hölper S, Krüger M, et al Genetic compensation induced by deleterious mutations but not gene knockdowns. Nature. 2015;0: 1–4. 10.1038/nature14580 26168398

[15] Swift MR, Pham VN, Castranova D, Bell K, Poole RJ, Weinstein BM. SoxF factors and Notch regulate nr2f2 gene expression during venous differentiation in zebrafish. Dev Biol. Elsevier; 2014;390: 116–125. 10.1016/j.ydbio.2014.03.018 PMC4104406 24699544

[16] van Impel A, Zhao Z, Hermkens DMA, Roukens MG, Fischer JC, Peterson-maduro J, et al Divergence of zebrafish and mouse lymphatic cell fate specification pathways. Development. 2014;141: 1228–1238. 10.1242/dev.105031 24523456 PMC3943180

[17] Wakayama Y, Fukuhara S, Ando K, Matsuda M, Mochizuki N. Cdc42 mediates Bmp—Induced sprouting angiogenesis through Fmnl3-driven assembly of endothelial filopodia in zebrafish. Dev Cell. Elsevier Inc.; 2015;32: 109–122. 10.1016/j.devcel.2014.11.024 25584797

[18] Phng LK, Gebala V, Bentley K, Philippides A, Wacker A, Mathivet T, et al Formin-mediated actin polymerization at endothelial junctions is required for vessel lumen formation and stabilization. Dev Cell. 2015;32: 123–132. 10.1016/j.devcel.2014.11.017 25584798

[19] Faucherre A, Kissa K, Nargeot J, Mangoni ME, Jopling C. Piezo1 plays a role in erythrocyte volume homeostasis. Haematologica. 2014;99: 70–75. 10.3324/haematol.2013.086090 PMC4007942 23872304

[20] Shmukler BE, Huston NC, Thon JN, Ni C-W, Kourkoulis G, Lawson ND, et al Homozygous knockout of the piezo1 gene in the zebrafish is not associated with anemia. Haematologica. 2015;100: 294–299. 10.3324/haematol.2015.132449 PMC4666336 26294733

[21] Dereeper A, Guignon V, Blanc G, Audic S, Buffet S, Chevenet F, et al Phylogeny.fr: robust phylogenetic analysis for the non-specialist. Nucleic Acids Res. 2008;36: 465–469. 10.1093/nar/gkn180 PMC2447785 18424797

[22] Felsenstein J. PHYLIP-Phylogeny Inference Package (Version 3.2). Cladistics. 1989;5: 163–166.

[23] Nusslein-Volhard C, Dahm R. Zebrafish: a practical approach Nusslein-Volhard2002: New York: Oxford University Press; 2002.

[24] Kimmel CB, Ballard WW, Kimmel SR, Ullman B, Schilling TF. Stages of Embryonic development of the zebrafish. Dev Dyn. 1995;203: 253–310. 10.1002/aja.1002030302 8589427

[25] Lawson ND, Weinstein BM. In vivo imaging of embryonic vascular development using transgenic zebrafish. Dev Biol. 2002/08/09. 2002;248: 307–318. Available: http://www.ncbi.nlm.nih.gov/pubmed/12167406 12167406 10.1006/dbio.2002.0711

[26] Cermak T, Doyle EL, Christian M, Wang L, Zhang Y, Schmidt C, et al Efficient design and assembly of custom TALEN and other TAL effector-based constructs for DNA targeting. Nucleic Acids Res. 2011;39: e82 10.1093/nar/gkr218 21493687 PMC3130291

[27] Huang P, Xiao A, Zhou M, Zhu Z, Lin S, Zhang B. Heritable gene targeting in zebrafish using customized TALENs. Nat Biotechnol. 2011;29: 699–700. 10.1038/nbt.1939 21822242

[28] Thisse C, Thisse B. High-resolution in situ hybridization to whole-mount zebrafish embryos. Nat Protoc. Nature Publishing Group; 2008;3: 59–69. Available: 10.1038/nprot.2007.514 18193022

[29] Cheng CN, Li Y, Marra AN, Verdun V, Wingert R a. Flat mount preparation for observation and analysis of zebrafish embryo specimens stained by whole mount in situ hybridization. J Vis Exp. 2014; e51604. 10.3791/51604 PMC4219405 25078510

[30] Detrich HW 3rd, Kieran MW, Chan FY, Barone LM, Yee K, Rundstadler JA, et al Intraembryonic hematopoietic cell migration during vertebrate development. Proc Natl Acad Sci U S A. 1995/11/07. 1995;92: 10713–10717. Available: http://www.ncbi.nlm.nih.gov/pubmed/7479870 7479870 10.1073/pnas.92.23.10713 PMC40682

[31] Eisen JS, Smith JC. Controlling morpholino experiments: don’t stop making antisense. Development. 2008/04/12. 2008;135: 1735–1743. 10.1242/dev.001115 18403413

[32] Palpant NJ, Pabon L, Rabinowitz JS, Hadland BK, Stoick-Cooper CL, Paige SL, et al Transmembrane protein 88: a Wnt regulatory protein that specifies cardiomyocyte development. Development. 2013;3808: 3799–3808. 10.1242/dev.094789 PMC3754478 23924634

[33] Novodvorsky P, Watson O, Gray C, Wilkinson RN, Reeve S, Smythe C, et al Klf2ash317 mutant zebrafish do not recapitulate morpholino-induced vascular and haematopoietic phenotypes. PLoS One. 2015;10. 10.1371/journal.pone.0141611 PMC4624238 26506092

[34] Nicoli S, Standley C, Walker P, Hurlstone A, Fogarty KE, Lawson ND. MicroRNA-mediated integration of haemodynamics and Vegf signalling during angiogenesis. Nature. 2010/04/07. 2010;464: 1196–1200. 10.1038/nature08889 20364122 PMC2914488

[35] Swift MR, Weinstein BM. Arterial-venous specification during development. Circ Res. 2009;104: 576–88. 10.1161/CIRCRESAHA.108.188805 19286613

[36] Stainier DYR, Kontarakis Z, Rossi A. Making sense of anti-sense data. Dev Cell. Elsevier Inc.; 2015;32: 7–8. 10.1016/j.devcel.2014.12.012 25584794

[37] Ivanov IP, Firth AE, Michel AM, Atkins JF, Baranov P V. Identification of evolutionarily conserved non-AUG-initiated N-terminal extensions in human coding sequences. Nucleic Acids Res. 2011;39: 4220–4234. 10.1093/nar/gkr007 21266472 PMC3105428

[38] Dabrowski M, Bukowy-Bieryllo Z, Zietkiewicz E. Translational readthrough potential of natural termination codons in eucaryotes—The impact of RNA sequence. RNA Biol. 2015;12: 950–958. 10.1080/15476286.2015.1068497 26176195 PMC4615788

[39] Kozak M. Pushing the limits of the scanning mechanism for initiation of translation. Gene. 2002;299: 1–34. 12459250 10.1016/S0378-1119(02)01056-9 PMC7126118

[40] Tikole S, Sankararamakrishnan R. A survey of mRNA sequences with a non-AUG start codon in RefSeq database. J Biomol Struct Dyn. 2006;24: 33–42. 10.1080/07391102.2006.10507096 16780373

[41] Bedell VM, Westcot SE, Ekker SC. Lessons from morpholino-based screening in zebrafish. Brief Funct Genomics. 2011;10: 181–188. 10.1093/bfgp/elr021 21746693 PMC3144740

[42] Robu ME, Larson JD, Nasevicius A, Beiraghi S, Brenner C, Farber S a, et al P53 Activation By Knockdown Technologies. PLoS Genet. 2007;3: e78 10.1371/journal.pgen.0030078 17530925 PMC1877875

[43] Ekker SC, Larson JD. Morphant technology in model developmental systems. Genesis. 2001;30: 89–93. 11477681 10.1002/gene.1038

[44] Gerety SS, Wilkinson DG. Morpholino artifacts provide pitfalls and reveal a novel role for pro-apoptotic genes in hindbrain boundary development. Dev Biol. Elsevier; 2011;350: 279–289. 10.1016/j.ydbio.2010.11.030 PMC3111810 21145318

[45] Chen J. Impaired cardiovascular function caused by different stressors elicits a common pathological and transcriptional response in zebrafish embryos. Zebrafish. 2013;10: 389–400. 10.1089/zeb.2013.0875 23837677 PMC3760051

[46] North TE, Goessling W, Peeters M, Li P, Ceol C, Lord AM, et al Hematopoietic stem cell development is dependent on blood flow. Cell. Elsevier Ltd; 2009;137: 736–48. 10.1016/j.cell.2009.04.023 PMC2722870 19450519

